# Lipocalin 10 as a New Prognostic Biomarker in Sepsis-Induced Myocardial Dysfunction and Mortality: A Pilot Study

**DOI:** 10.1155/2021/6616270

**Published:** 2021-05-22

**Authors:** Lu Wang, Wenjie Xie, Guang Li, Bo Hu, Wei Wu, Liying Zhan, Handong Zou

**Affiliations:** ^1^Department of Critical Care Medicine, Renmin Hospital of Wuhan University, Wuhan 430060, China; ^2^Department of Ultrasound Imaging, Renmin Hospital of Wuhan University, Wuhan 430060, China

## Abstract

**Introduction:**

Sepsis-induced myocardial dysfunction (SIMD) is the most common complications of sepsis and septic shock with extremely high incidence and mortality. Lipocalin 10 (Lcn10) has recently been identified as a potential biomarker for heart failure, yet its relation to sepsis has not been investigated. The purpose of this study was to explore whether circulating Lcn10 could be used as a prognostic tool in patients with SIMD.

**Methods:**

In this single-center observational pilot study, seventy-five sepsis patients were enrolled after sepsis diagnosis or ICU admission (45.3% female, median age 60 years), and 35 patients (46.7%) developed myocardial dysfunction. Serum Lcn10 levels of septic patients were measured using the enzyme-linked immunosorbent assay (ELISA) at the time of admission. Other biomarkers of cardiac function and Lcn10 concentration were compared between SIMD and non-SIMD groups.

**Results:**

We observed that the median Lcn10 levels were 2.780 ng/mL in patients with SIMD and 2.075 ng/mL in patients without SIMD (*P* < 0.05). The area under the receiver operating characteristic (ROC) curve for the diagnosis of SIMD was 0.797 (*P* < 0.05). In addition, elevated serum Lcn10 levels at the time of admission were positively associated with 28-day mortality in septic patients.

**Conclusions:**

Our study indicates that circulating Lcn10 levels may serve as a novel biomarker for the diagnosis and prognosis of myocardial dysfunction induced by sepsis. An additional large multicenter study may be warranted to confirm the findings of this study.

## 1. Introduction

Sepsis is a life-threatening syndrome arising from a dysregulated host response to infection; the incidence of sepsis has increased by more than 30% in recent years [1, 2]. Myocardial injury is one of the most common complications of sepsis. Sepsis-induced myocardial dysfunction (SIMD) was first reported in 1984 by Parker et al. [3]. Nearly 50% of sepsis patients experience cardiac failure, which increases the risk of mortality to 68% [4]. Previous studies revealed that SIMD is mainly caused by myocardial depressant factors such as TNF*α*, interleukin-1 (IL-1), IL-6, and lipopolysaccharide (LPS) and downstream inflammatory mediators, including platelet activating factor and nitric oxide (NO) [5, 6]. Early diagnosis and prediction of outcomes in sepsis patients with SIMD remain difficult in clinical settings. Recently, there have been advancements in the discovery of biomarkers that could potentially improve the diagnosis of sepsis and identify patients with severe sepsis. Classic cardiac biomarkers, including N-terminal pro-b-type natriuretic peptide (NT-proBNP) and troponin I (TnI), may have some predictive value for the diagnosis and prognosis of SIMD [7–9]. However, the extent to which these markers can be utilized in the early diagnosis or prediction of SIMD is poorly understood due to varying and typically complex clinical signs. Given that early diagnosis of SIMD may lead to less severe cardiovascular events [5], the search for a novel biomarker is critical to improve the survival rate of SIMD patients.

Lipocalin (LCN) family proteins are evolutionarily conserved small proteins (18-40 kDa) [10]. They are expressed in numerous tissues and play important roles in multiple cellular processes (i.e., inflammation, detoxification, and immune activation) by transporting small hydrophobic molecules such as steroids, bilins, retinoids, and lipids to target cells [11]. In humans, several LCN family proteins have been extensively studied as biomarkers for SIMD [12, 13]. For example, H-FABP, a lipocalin family member, has demonstrated potential value as a diagnostic marker for SIMD with a sensitivity of 83% and specificity of 73% [12]. Elevated Lcn2 (also referred to as NGAL) in sepsis patients was found to be related to cardiac injury and positively correlated with BNP levels [13]. Lcn10 consists of an 8-stranded antiparallel beta-barrel that develops a cup-shaped ligand-binding pocket or cavity similar to other lipocalins [14]. Of specific interest, Lcn10 is highly expressed in the heart, lymph node, spleen, and thyroid [15]. Utilizing an RNA-seq approach, di Salvo et al. indicated that Lcn10 expression was significantly reduced in patients with heart failure and right ventricular dysfunction [16]. Moreover, a meta-analysis found that Lcn10 gene expression was downregulated in three individual analyses of human left ventricle tissues from patients with dilated cardiomyopathy [17]. This finding prompted us to investigate the possible relationship between Lcn10 and myocardial dysfunction induced by sepsis.

Considering the potential role of Lcn10 in heart failure, we hypothesized that serum Lcn10 levels may adaptively contribute to the pathogenesis of sepsis in patients who have cardiac dysfunction. As such, the current study investigated serum Lcn10 levels in sepsis patients at the time of admission to characterize the possible correlation between Lcn10 levels and sepsis-induced myocardial dysfunction. We sought to develop a sensitive, precise, and specific biomarker for the assessment of SIMD.

## 2. Methods

### 2.1. Study Population

The protocol of the study conformed to the ethical guidelines of the 2008 Helsinki Declaration. Renmin Hospital of Wuhan University guaranteed appropriate ethical and bioethical procedures and certificated this study (no. WDRY2019-K027). After approval of the study, all potential septic patients in ICU at the admission and 20 healthy donors were enrolled. Subjects in the control group were healthy donors who were recruited in the Medical Examination Center. All participants or their authorizer signed informed consent forms before the study. Screening criteria for sepsis performed by the attending physician is suspected infection and qSOFA ≥ 2. We identified 96 patients primarily diagnosed with suspected sepsis or septic shock by clinical and laboratory investigations from April 2018 to September 2019 at Renmin Hospital of Wuhan University.

The inclusion criteria were as follows: (1) meeting the diagnostic criteria for Sepsis-3 developed by the American Society of Critical Care Medicine/European Society of Intensive Care Medicine (note: septic shock refers to sepsis patients who required a vasopressor in order to maintain a mean arterial blood pressure greater than 65 mmHg and a serum lactate level higher than 2  mmol/L after sufficient fluid resuscitation) [2, 4]; (2) admission into the intensive care unit (ICU) by the emergency department (ED); and (3) age between 18 and 80 years old. Exclusion criteria were as follows: (1) age < 18 years or >80 years (*n* = 3); (2) previously diagnosed with chronic heart, kidney and liver failure, and malignant cancers (*n* = 8); (3) not diagnosed with sepsis finally (*n* = 5); (4) failing to complete all the tests outlined in the study (*n* = 2); and (5) dropping out during 28-day follow-ups excluding death (*n* = 3).

In this study, diagnosis of sepsis-induced myocardial dysfunction followed the criteria used in Mayo Clinic which include ejection fraction (EF) < 50%, high‐sensitivity TnI (hs‐TnI) > 0.78 ng/mL (normal range at Renmin Hospital is 0-0.78 ng/mL) or NT − proBNP > 500 pg/mL in the first 24 h of ICU admission [18]. Echocardiograms (ECGs) were performed by the Department of Medical Ultrasonic of Renmin Hospital.

### 2.2. Clinical Design

On enrollment, the team for this study created standardized case report forms (CRF) to record every patient's characteristic data. At the time of the ICU admission, clinical data were recorded, including demographic characteristics, past medical history, vital signs, physical examination results, laboratory data, electrocardiogram (ECG), and imaging data. Blood samples were collected for white blood cell count, blood biochemistry, arterial blood gas, blood cultures, and urine cultures at the same time point by the laboratory center of the hospital. The cardiac index (CI) was detected by using Lifegard ICG Hemodynamic Monitors (Analogic, USA). Additionally, we checked the vasopressor usage and hospital length of stay from patients' medical charts. We calculated Acute Physiology, Age and Chronic Health Evaluation II (APACHE) scores and Sequential Organ Failure Assessment (SOFA) scores after all data were collected [19]. The 28-day follow-up was conducted by a telephone survey with the aim of assessing mortality.

### 2.3. Serum Collection and Measurement of Lcn10 Levels

After entering ICU, we collected patients' blood samples at the same time as other blood tests before the administration of any treatment. Then, samples were placed at room temperature for 30 min followed by centrifugation at 3000 rpm for 15 min at 4°C. The serum samples were stored at -80°C until further analysis. Lcn10 levels in the collected serum samples were measured in duplicate by ELISA kits from Elabscience Biotechnology Co., Ltd. (Wuhan, China) according to the manufacturer's instructions. A Multiskan Mk3 microplate reader (Thermo Scientific, USA) was utilized to analyze absorbance at 450 nm of each sample.

### 2.4. Statistical Analysis

Data were analyzed using the Statistical Package for the Social Sciences (ver 19.0) or GraphPad Prism 7 (GraphPad Software), and *P* values < 0.05 were determined statistically significant. Continuous parameters were presented as median with interquartile ranges (IQR) and analyzed by the Mann-Whitney *U* test to determine significance. Gender and death before day 28 were presented as frequency and percentages. These data were analyzed by the chi-square (*χ*^2^) test to determine significance between the cohorts for categorical variables. Serum Lcn10 levels were summarized by non-SIMD and SIMD using boxplots with a summary of the median, quartiles, range, and extreme values. A multivariate Cox regression analysis was performed adjusting for important clinical parameters including age, gender, and BMI.

To determine diagnostic values, we assessed ROC curves and areas under the receiver operating characteristic curves (AUCs) to discriminate SIMD from sepsis and predict 28-day mortality. The best cut-off value was identified as serum Lcn10 levels present the greatest total of sensitivity and specificity by the Youden index (*J*) method [20]. The positive predictive value (PPV) and negative predictive value (NPV) were calculated as follows:

(1) $$\begin{array}{c} \text{PPV}=\frac{\text{True Positive}}{\text{True Positive}+\text{False Positive}}\times 100.\\ \text{NPV}=\frac{\text{True Negative}}{\text{True Negative}+\text{False Negative}}\times 100. \end{array}$$

Differences between the curves were assessed using the log-rank test. A two-sided significance level of 0.05 was used for statistical inference, and all statistical tests were two-tailed.

## 3. Results

### 3.1. Demographics and Clinical Details of Septic Patients

**Figure 1:**
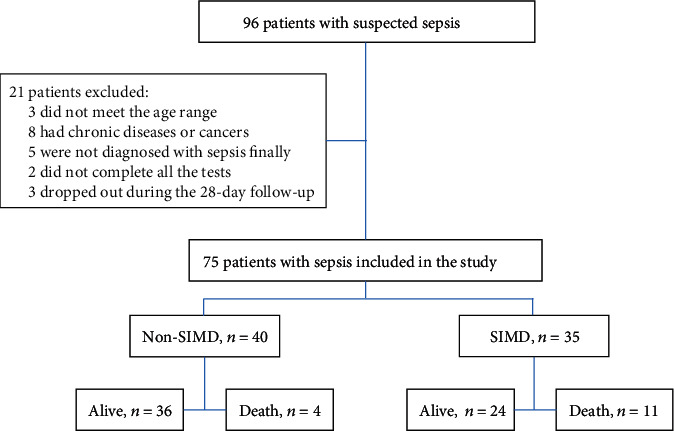
Flow diagram and outcome of the study participants. SIMD: sepsis-induced myocardial dysfunction.

**Figure 2:**
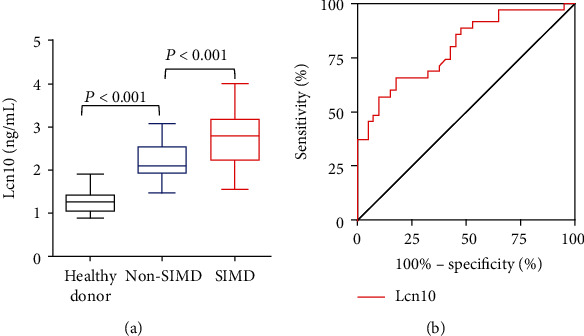
Diagnostic value of Lcn10 in sepsis-induced myocardial dysfunction. (a) Serum Lcn10 levels in the healthy donors, SIMD group, and non-SIMD group. (b) Receiver operator characteristic curves of Lcn10 for the diagnosis of SIMD. The AUC of Lcn10 for the diagnosis of SIMD in septic patients was 0.797 (*P* < 0.001; 95% confidence interval, 0.696-0.897).

This prospective study screened a total of 96 patients who were admitted to the ICU, 75 of them were enrolled in the final study (Figure 1). The median age of all patients was 60 years (IQR 55-68 years), and 54.7% of the participants were male. The median length of stay in the ICU for all septic patients was 8 days (IQR 6-11 days). Among 75 sepsis patients included in this study, we identified 35 patients (46.7%) with myocardial dysfunction (SIMD). There were no significant differences in diastolic blood pressure, heart rate, levels of C-reaction protein (CRP), procalcitonin (PCT), CK-MB, and myoglobin between the SIMD group and non-SIMD group (*P* > 0.05). However, the 28-day mortality of the SIMD group was 31.4%, which was significantly higher than that of the non-SIMD group (*P* = 0.021; Table 1). Moreover, patients with myocardial dysfunction performed poorly in many other clinical assessments including systolic blood pressure, APACHE II score, SOFA score, hs-TnI, and lactate levels (*P* < 0.05; Table 1). On admission, septic patients exhibited significantly higher serum Lcn10 level compared with healthy donors (2.370 ng/mL *vs.* 1.257 ng/mL, *P* < 0.001; Figure 2(a)). Importantly, our analysis results showed that median serum Lcn10 levels were significantly higher in the SIMD group than in the non-SIMD group (2.780 ng/mL *vs*. 2.075 ng/mL, *P* < 0.001; Figure 2(a)). The significant differences in Lcn10 levels remained unchanged after adjusting for age, gender, and BMI.

### 3.2. Diagnostic Value of Sepsis-Induced Myocardial Dysfunction

Given that sepsis patients with myocardial dysfunction exhibited higher levels of circulating Lcn10, we next hypothesized that Lcn10 might be a good prognostic marker of SIMD. To this end, ROC curves were generated for Lcn10 for discriminating SIMD with significant *P* values (Figure 2(b)), and the AUC of Lcn10 was 0.797 (95% CI 0.696-0.897, *P* < 0.001). The sensitivities and specificities of Lcn10 as a diagnostic marker of SIMD at various cut-off levels of serum Lcn10 are shown in Table 2, and the optimal cut-off was 2.664 ng/mL. Accordingly, PPV was 76.67% and NPV was 73.33%.

### 3.3. Predictive Value of Lcn10 for 28-Day Mortality in Septic Patients

**Figure 3:**
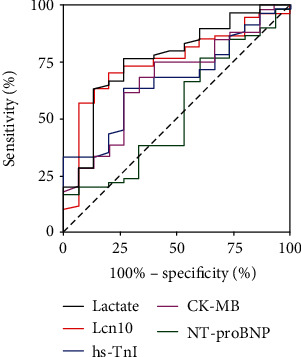
Predictive performance of Lcn10 in 28-day mortality of SIMD patients. Compared to other commonly used biomarkers, ROC curves showed that Lcn10 is a more powerful predictor for 28-day mortality of SIMD patients. The AUC was 0.753 (*P* = 0.003; 95% confidence interval, 0.624-0.882).

By ROC curve analysis, we observed that Lcn10 showed stronger power than other cardiac biomarkers in the prediction of 28-day mortality in septic patients. The area under the ROC curve of Lcn10 was 0.753 (*P* = 0.003) with the optimal cut-off of 2.286 ng/mL, PPV was 35.0%, and NPV was 97.1%. In comparison, AUROC of lactate, hs-TnI, NT-proBNP, and CK-MB was 0.766 (*P* = 0.002), 0.667 (*P* = 0.046), 0.673 (*P* = 0.039), and 0.531 (*P* = 0.711), respectively (Figure 3). These analysis results demonstrate that circulating Lcn10 level may serve as an independent risk factor for mortality in patients with sepsis. Thus, serum Lcn10 concentration should have great potential as a novel biomarker to predict the 28-day mortality in septic patients.

## 4. Discussion

In the present study, we observed elevated serum levels of Lcn10 on admission in sepsis patients with myocardial dysfunction. The mechanism of Lcn10 in SIMD has not yet been illustrated. Scheraga et al. reported that Lcn10 expression could be synergistically upregulated by wound and heat shock proteins, which were characterized as acute-phase proteins after infection [21]. Lcn10 was also found to be T-reg cell specific, proving its potential role in the innate immune response [22]. Moreover, high expression of Lcn10 in human subcutaneous and epigastric tissues suggests that it plays a role in lipid metabolism [23]. Lcn10 has been shown to be regulated by the Notch pathway in B cells, which needs to be confirmed with deeper research [24]. Thus, we speculate that Lcn10, like Lcn2 [25], might be stimulated by inflammation or infection playing a role in the regulation of acute cardiac injury.

To our knowledge, this is the first prospective clinical study to assess the diagnostic and prognostic value of serum Lcn10 for SIMD. In the present study, we observed that the incidence and mortality rates were consistent with those of previous studies [26, 27]. We found elevated serum Lcn10 levels on admission in sepsis patients with myocardial dysfunction. Surprisingly, according to the AUROC analysis, Lcn10 showed a higher prognostic value for 28-day mortality than hs-TnI, NT-proBNP, and CK-MB. Our findings suggest that the level of serum Lcn10 is a biomarker for the prognosis of sepsis, especially SIMD, and is worthy of further study.

However, our findings in patients were different from previous findings based on RNA sequencing data, which showed a dramatic decrease in Lcn10 RNA levels in the human right ventricle (heart failure) and left ventricle (dilated cardiomyopathy) [16, 17]. We propose the following explanations for this discrepancy. The difference may be due to the different disease stages of the patients, the area from which the samples were collected, or the different immune statuses of patient cardiomyocytes. Another reason is that serum Lcn10 is secreted at different levels by different cardiac tissues. In addition, the current study utilized a definition of SIMD which combined LVEF, troponin, and NT-proBNP and could not elucidate the complex cardiac function in sepsis, including right ventricular function.

At present, classic cardiac biomarkers (i.e., NT-proBNP, troponin I, and CK-MB) are usually used as auxiliary tools for the diagnosis and prognosis of SIMD [9, 28]. The current study showed Lcn10 is a new biomarker for the diagnosis of SIMD. The optimal cut-off of 2.664 ng/mL could be used as a diagnostic tool for SIMD as the sensitivity of 65.7% and specificity of 82.5% are much higher than those of hs-TnI, which has a sensitivity of 58.6% and specificity of 59.1% [8, 29]. Importantly, our data showed that the ability of Lcn10 to predict mortality was superior to that of hs-TnI, NT-proBNP, and CK-MB, as demonstrated by the AUC. Lcn10 may be a more powerful predictor for 28-day mortality than conventional biomarkers (Figure 3). These results were similar to those of previous studies on Lcn2, which showed that Lcn2 levels were increased and positively correlated with BNP and NT-proBNP in HIV infection and acute heart failure [30, 31].

In addition, the definition of SIMD includes the left ventricular systolic dysfunction, diastolic dysfunction, and right ventricular dysfunction induced by sepsis. Indeed, left ventricular ejection fraction (LVEF), LV end-diastolic volume (LVEDV), and the ratio of *E*/*e*′ measured by echocardiography are typical parameters that are significantly related to hypotension and cardiac injury and are usually used as diagnostic indicators of SIMD [32, 33]. However, due to different equipment and criteria and the inevitable interobserver variability among operators, neither systolic dysfunction nor diastolic dysfunction has been confirmed to be associated with mortality [34]. Therefore, due to the lack of echocardiography in resource-limited settings, the determination of serum Lcn10 levels will be more cost-effective than echocardiography because the measurement only requires a well-developed ELISA method.

Finally, there are a couple of shortcomings in this study. First, in the absence of a gold standard for diagnosing SIMD, a definition based on the combination of LVEF and hs-TnI or NT-proBNP was developed. As already stated, LVEF only represents the contraction function of the heart. As the understanding of SIMD is limited, the more complex aspects of SIMD remain to be uncovered. Second, due to limited access to medical records, a more detailed analysis of cardiac function could not be performed. Finally, this study was conducted at a single center with a small sample size, and the time of parameter collection was limited. However, the wealth of pilot data in this study could be used as the basis for subsequent multicenter studies.

In conclusion, this clinical observation demonstrates that the level of serum Lcn10 in patients with sepsis complicated with cardiac injury is higher than that in non-SIMD sepsis patients when they are admitted to the hospital. The serum Lcn10 level is positively correlated with the incidence of myocardial dysfunction caused by sepsis. Remarkably, Lcn10 is a more reliable predictor of 28-day mortality than other commonly used biomarkers. Our results strongly suggest the practicability of serum Lcn10 levels as a potential predictor of SIMD. To validate the relationship between the level of serum Lcn10 and sepsis-induced myocardial dysfunction, further prospective investigations are warranted.

## Figures and Tables

**Table 1:** Demographic and clinical data of the study population. Values expressed in percentages (%) indicate the proportion of patients within each cohort for each variable. Data are presented as median with interquartile ranges (IQR) where specified. SIMD: sepsis-induced myocardial dysfunction; BMI: body mass index; BP: blood pressure; CRP: c-reactive protein; PCT: procalcitonin; APACHE: Acute Physiology: Age and Chronic Health Evaluation; SOFA: Sequential Organ Failure Assessment; NT-proBNP: N-terminal pro-b-type natriuretic peptide; hs-TnI: high-sensitivity troponin I; CK-MB: creatine kinase-MB; Lcn10: lipocalin 10. ^∗^*P* < 0.05.

Variables	Non-SIMD (*n* = 40)	SIMD (*n* = 35)	*P* value
Age (y), median (IQR)	62.5 (55.3-68.8)	58.0 (53.0-67.0)	0.189
Gender, male, *n* (%)	20 (50%)	21 (60%)	0.386
BMI (kg/m^2^), median (IQR)	22.0 (20.0-25.75)	20.0 (19.0-22.0)	0.071
Hospital length of stay (days), median (IQR)	7 (5-12)	7 (6-11)	0.905
Death before day 28, *n* (%)	4 (10%)	11 (31.4%)	0.021^∗^
Systolic BP (mmHg), median (IQR)	97 (86-119)	90 (80-97)	0.046^∗^
Diastolic BP (mmHg), median (IQR)	54 (44-64)	52 (45-62)	0.864
Heart rate (beat/min), median (IQR)	91 (82-110)	93 (84-112)	0.381
Lactate (mmol/L), median (IQR)	1.225 (0.890-2.175)	3.760 (2.330-5.890)	<0.001^∗^
CRP (mg/L), median (IQR)	149 (99-203)	156 (89-244)	0.737
PCT (ng/mL), median (IQR)	4.76 (1.27-15.30)	9.43 (2.18-79.34)	0.359
APACHE II score, median (IQR)	17 (13-22)	21 (15-26)	0.015^∗^
SOFA score, median (IQR)	7 (5-10)	11 (9-14)	<0.001^∗^
NT-proBNP (pg/mL), median (IQR)	442 (208-2129)	983 (534-2310)	0.053
hs-TnI (ng/mL), median (IQR)	0.328 (0.151-1.854)	1.030 (0.540-2.780)	0.004^∗^
CK-MB (ng/mL), median (IQR)	5.74 (3.19-12.07)	11.71 (3.08-19.65)	0.350
Myoglobin (*μ*g/L), median (IQR)	154 (89-347)	207 (144-456)	0.057

**Table 2:** Sensitivity and specificity of Lcn10 levels for diagnosis of SIMD.

Lcn10 cut-off (ng/mL)	Sensitivity (%)	Specificity (%)
>1.469	100.0	2.5
>2.664	65.7	82.5
>3.981	2.9	100.0

## Data Availability

The underlying data supporting the results are all from our clinical data.
